# Effect of Thrombolysis on Circulating Microparticles in Patients with ST-Segment Elevation Myocardial Infarction

**DOI:** 10.1155/2023/5559368

**Published:** 2023-11-18

**Authors:** Zhe Li, Wei Zhang, Qun-Rang Wang, Yu-juan Yang, Xin-Hong Liu, Gong Cheng, Feng-Jun Chang

**Affiliations:** ^1^Department of Cardiology, Shaanxi Provincial People's Hospital, Xi'an, China; ^2^Department of Cardiology, Affiliated Hospital of Shaanxi University of Chinese Medicine, Xian'yang, China

## Abstract

**Objective:**

We demonstrated that circulating microparticles (MPs) are increased in patients with coronary heart disease (both chronic coronary syndrome (CCS) and acute coronary syndrome). Whether thrombolysis affects MPs in patients with ST-segment elevation myocardial infarction (STEMI) with or without percutaneous coronary intervention (PCI) is unknown.

**Methods:**

This study was divided into three groups: STEMI patients with thrombolysis (*n* = 18) were group T, patients with chronic coronary syndrome (*n* = 20) were group CCS, and healthy volunteers (*n* = 20) were the control group. Fasting venous blood was extracted from patients in the CCS and control groups, and venous blood was extracted from patients in the T group before (pre-T) and 2 hours after (post-T) thrombolysis. MPs from each group were obtained by centrifugation. After determining the concentration, the effects of MPs on endothelial nitric oxide synthase (eNOS) and inducible nitric oxide synthase (iNOS) in rat myocardial tissue in vitro were detected by immunohistochemistry and western blotting. Changes in nitric oxide (NO) and oxygen free radicals (O_2_^•–^) were also detected. The effect of MPs on vasodilation in isolated rat thoracic aortae was detected.

**Results:**

Compared with that in the control group (2.60 ± 0.38 mg/ml), the concentration of MPs was increased in patients with CCS (3.49 ± 0.72 mg/ml) and in STEMI patients before thrombolysis (4.17 ± 0.58 mg/ml). However, thrombolysis did not further increase MP levels (post-T, 4.23 ± 1.01 mg/ml) compared with those in STEMI patients before thrombolysis. Compared with those in the control group, MPs in both CCS and STEMI patients before thrombolysis inhibited the expression of eNOS (both immunohistochemistry and western blot analysis of phosphorylation at Ser1177), NO production in the isolated myocardium and vasodilation in vitro and stimulated the expression of iNOS (immunohistochemistry and western blot analysis of phosphorylation at Thr495), and the generation of O_2_^•–^ in the isolated myocardium. The effects of MPs were further enhanced by MPs from STEMI patients 2 hours after thrombolysis.

**Conclusion:**

Changes in MP function after thrombolysis may be one of the mechanisms leading to ischemia–reperfusion after thrombolysis.

## 1. Introduction

ST-segment elevation myocardial infarction (STEMI), which is mainly manifested as myocardial necrosis consequent to an ischemic injury with persistent ST-segment elevation on the electrocardiogram, is the most common acute cardiovascular disease and also the major causes of mortality worldwide [1]. Percutaneous coronary intervention (PCI) is an effective treatment for STEMI revascularization, but many primary hospitals have no conditions for PCI (or patients refuse PCI). Thrombolysis, especially the immediate effect of early and timely thrombolysis, has a therapeutic effect that is similar to that of direct PCI [2, 3]. However, complications after thrombolysis, especially ischemia–reperfusion injury (IRI), seriously affect the prognosis of STEMI. Circulating microparticles (MPs) are nanoscale particles released by endothelial cells, monocytes, and platelets in response to various stimuli. Originally, MPs were considered useless “cell garbage.” Both we and other researchers [4–8] found that MPs have multiple molecular functions and participate in a variety of biological processes of cardiovascular disease related to endothelial function, coagulation, oxidative stress, and inflammation. The main mechanism of IRI is the massive production of reactive oxygen free radicals [9], which is one of the functions of MPs. However, it has not been reported whether the function and quantity of MPs are affected by thrombolysis or whether MPs participate in IRI after thrombolysis. In the present study, we examined the function and quantity of MPs in STEMI patients before and after thrombolysis and explored the mechanism by which MPs participate in IRI after thrombolysis.

## 2. Materials and Methods

### 2.1. Study Population

STEMI patients (PCI was refused by them or their families, *n* = 18) receiving thrombolytic therapy and patients with chronic coronary syndrome (CCS, *n* = 20) in the Department of Cardiology, Affiliated Hospital of Shaanxi University of Traditional Chinese Medicine, from 2021/01 to 2021/12 were recruited. Patients with diseases that may affect MPs were excluded, including diabetes, severe trauma, infectious disease, hypertension, renal failure, multiple sclerosis, lupus anticoagulant, or acute rheumatic diseases. Twenty age- and sex-matched healthy subjects were recruited as a control group. All subjects signed informed consent forms, and our study was approved by the Ethics Committee of the Affiliated Hospital of Shaanxi University of Traditional Chinese Medicine.

### 2.2. MP Isolation

Venous blood samples (fasting venous blood from patients in the CCS and control groups, venous blood from STEMI patients before (pre-T) and 2 hours after (post-T) thrombolysis) were collected, and MPs were obtained by centrifugation (Beckman, CA, USA) as follows [7]. After centrifugation (11 000 g, 4°C, 2 min), the upper plasma (platelet-poor plasma) was obtained. Then, MPs (precipitate in the bottom of the centrifuge tube) were collected from platelet-poor plasma by centrifugation (13 000 g, 4°C, 45 min). Finally, the MPs were resuspended in RPMI 1640 (Gibco, Invitrogen, Carlsbad, CA, 100 *μ*l) and consumed within 3 weeks (stored at -80°C). Because the MPs were largely consumed in follow-up experiments and the blood samples were limited, we pooled MPs from patients within the same group.

### 2.3. Immunohistochemistry

Immunohistochemistry was performed [10]. MPs (3 mg) from each group or RPMI were injected into male Sprague-Dawley (SD) rats through the dorsal vein of the penis. Six hours later, the heart was fixed, dehydrated, paraffin-embedded, and sectioned. The expression of endothelial nitric oxide synthase (eNOS, United States, Abcam) and inducible nitric oxide synthase (iNOS, United States, Abcam) was detected by immunohistochemistry (streptavidin peroxidase (SP) method) using a DBA kit (China, Shanghai Yaji Biotechnology).

### 2.4. Superoxide (O_2_^•–^) Detection

MPs (3 mg) from each group or RPMI were injected into male SD rats through the dorsal vein of the penis. Six hours later, the thoracic aorta was separated and washed with the Krebs buffer (Sigma-Aldrich, 131 mM NaCl, 5.6 mM KCl_,_ 25 mM NaHCO_3_, 1 mM NaH_2_PO_4_H_2_O, 1 mM HEPES, 5 mM glucose, 2.5 mM CaCl_2_, 1 mM MgCl_2_, 100 *μ*M L-arginine, and pH 7.4). Then, the Krebs buffer with lucigenin (5 *μ*M, Sigma-Aldrich) was added and incubated in the dark for 5 minutes at room temperature. Finally, O_2_^•–^ was detected with a SpectraMax M5/M5e multidetection reader (Molecular Devices, CA, USA). The dry weight of aortic samples was obtained to determine O_2_^•–^ levels (*μ*g/mg protein).

### 2.5. Nitric Oxide (NO) Detection

MPs (3 mg) from each group or RPMI were injected into male SD rats through the dorsal vein of the penis. Six hours later, the heart was harvested. Then, NO levels were detected according to the nitric oxide kit instructions (China, Nanjing Jiancheng Biotechnology). The dry weight of aortic samples was obtained to determine NO levels (*μ*mol/g protein).

### 2.6. Western Blot Analysis

MPs (3 mg) from each group or RPMI were injected into male SD rats through the dorsal vein of the penis. Six hours later, the heart proteins were harvested to detect the expression of iNOS and eNOS and their phosphorylation by western blotting.

### 2.7. Vasodilatation Testing

MPs (3 mg) from each group or RPMI were injected into male SD rats through the dorsal vein of the penis. Six hours later, the thoracic aorta was isolated and placed in precooled Krebs buffer. After adipose tissue was removed, the thoracic aorta was cut into 3-5 mm thick vascular rings. Then, the rings were connected to an isometric force transducer (ADInstruments Co, Australia) and placed in the Krebs solution containing 5% CO_2_ and 95% O_2_ at 37°C for 30 min. Then, aortic ring stabilization was tested with KCl (60 mmol/l) at least three times. After being incubated with MPs for 30 min, the rings were preconstricted with phenylephrine (PE, Sigma-Aldrich, 10^−6^ mol/l). Immediately, acetylcholine (Ach: 10^−8^–10^−4^ mol/l, Sigma-Aldrich) was added to detect endothelium-dependent relaxation.

### 2.8. Statistical Analysis

All data were analyzed by SPSS 22.0 software and graphed with GraphPad Prism 5.0 software. All data are listed as the mean ± standard deviation. Independent-sample *t*-tests were used for comparisons between two groups, and one-way analysis of variance was used for multigroup comparisons. Differences were considered significant when *P* < 0.05.

## 3. Results

### 3.1. Clinical Data

All clinical characteristics of the controls and patients with CCS or STEMI are listed in Table 1.

### 3.2. Plasma MP Concentrations

**Figure 1:**
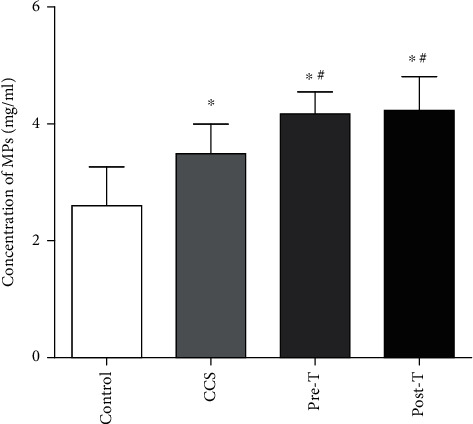
Microparticles (MPs) are increased in STEMI patients with or without thrombolysis. MPs were increased in both CCS patients (3.49 ± 0.72 mg/ml, *n* = 20) and STEMI patients before thrombolysis (pre-T, 4.17 ± 0.58 mg/ml, *n* = 18) compared with those in the control group (2.60 ± 0.38 mg/ml, *n* = 20). Thrombolysis did not further increase MP levels (post-T, 4.23 ± 1.01 mg/ml, *n* = 18). The data are means ± SDs; ^∗^ vs. control; ^#^ vs. CCS, *P* < 0.05.

Compared with those in the control group (2.60 ± 0.38 mg/ml, *n* = 20), plasma MP concentrations were slightly elevated in CCS patients (3.49 ± 0.72 mg/ml, *n* = 20). While plasma MP concentrations were significantly increased in STEMI patients before thrombolysis (pre-T, 4.17 ± 0.58 mg/ml, *n* = 18), thrombolysis did not further increase MP levels (post-T, 4.23 ± 1.01 mg/ml, *n* = 18) (Figure 1).

### 3.3. Effect of the MPs on eNOS and iNOS, as Determined by Immunohistochemistry

**Figure 2:**
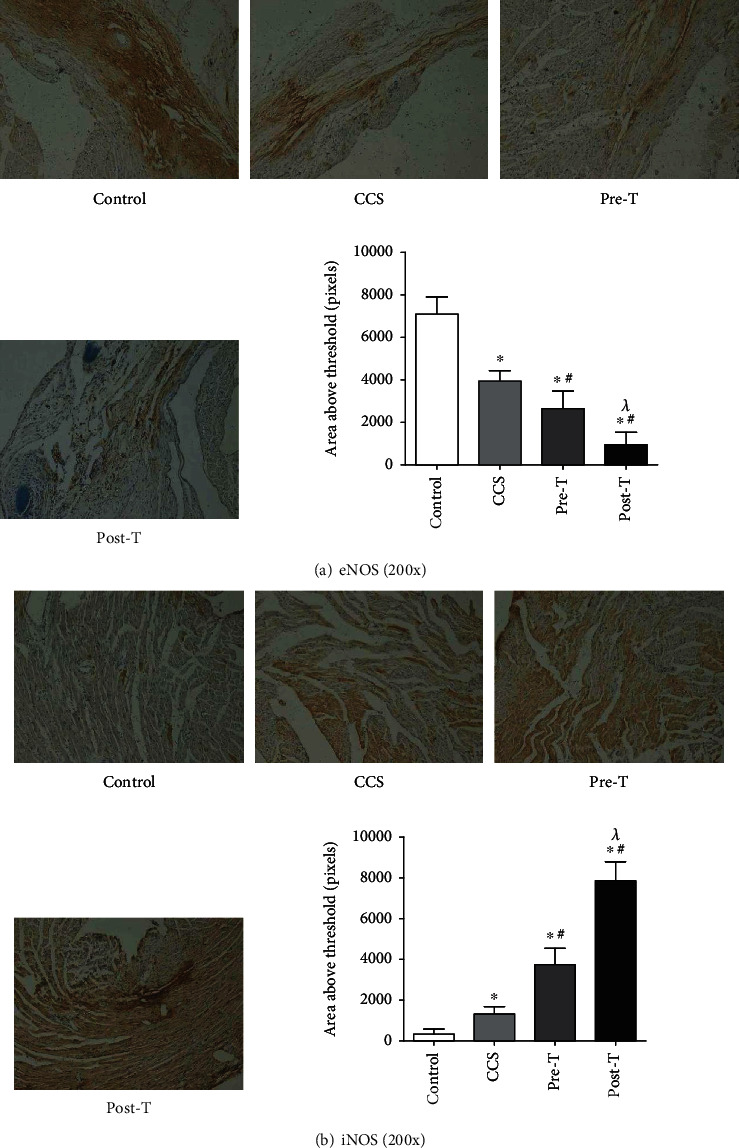
Effects of MPs from STEMI patients with or without thrombolysis on eNOS and iNOS expressions, as determined by immunohistochemistry. (a) Compared with those in the controls, MPs from CCS patients and STEMI patients before thrombolysis (pre-T) decreased the expression of eNOS in rat hearts. MPs from STEMI patients 2 hours after thrombolysis (post-T) further decreased the expression of eNOS in rat hearts. (b) Compared with those in the controls, MPs from both CCS patients and STEMI patients before thrombolysis (pre-T) increased the expression of iNOS in rat hearts. MPs from STEMI patients 2 hours after thrombolysis (post-T) further increased the expression of iNOS in rat hearts. The data are means ± SDs; ^∗^ vs. control; ^#^ vs. CCS; ^*λ*^ vs. pre-T, *P* < 0.05.

Compared with that in the control group (Figure 2(a)), MPs from CCS patients slightly decreased the expression of eNOS in the rat heart, as determined by immunohistochemistry (Figure 2(a)). MPs from STEMI patients before thrombolysis (pre-T, Figure 2(a)) decreased the expression of eNOS in the rat heart, as determined by immunohistochemistry, and the expression of eNOS in the rat heart was further decreased by MPs from STEMI patients 2 hours after thrombolysis (post-T, Figure 2(a)). The change in the expression of iNOS (Figure 2(b)) was the opposite to that of eNOS.

### 3.4. Effects of MPs on NO and O_2_^•−^ Generation

**Figure 3:**
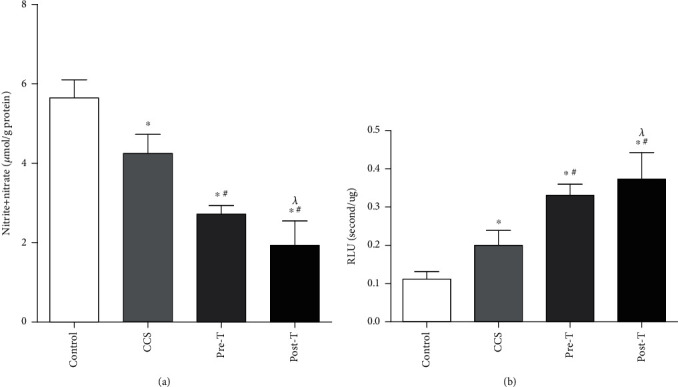
Effects of MPs from STEMI patients with or without thrombolysis on NO and O2^•−^ generation. (a) Compared with those in the controls, MPs from CCS patients and STEMI patients before thrombolysis (pre-T) decreased the generation of NO in rat hearts. MPs from STEMI patients 2 hours after thrombolysis (post-T) further decreased the generation of NO in rat hearts. (b) Compared with those in controls, MPs from CCS patients and STEMI patients before thrombolysis (pre-T) increased the production of O2^•−^ in rat hearts. MPs from STEMI patients 2 hours after thrombolysis (post-T) further increase the production of O2^•−^ in rat hearts. The data are means ± SDs; ^∗^ vs. control; ^#^ vs. CCS; ^*λ*^ vs. pre-T, *P* < 0.05.

The effects of MPs on NO and O_2_^•−^ generation were detected to investigate whether oxidative stress was activated. Compared with MPs from the control group, MPs from CCS and STEMI patients before thrombolysis slightly decreased NO (Figure 3(a)) production but increased O_2_^•−^ generation (Figure 3(b)). Moreover, the effect on NO and O_2_^•−^ generation was enhanced by MPs from STEMI patients 2 hours after thrombolysis (Figure 3).

### 3.5. Effects of MPs on eNOS and iNOS Expressions

**Figure 4:**
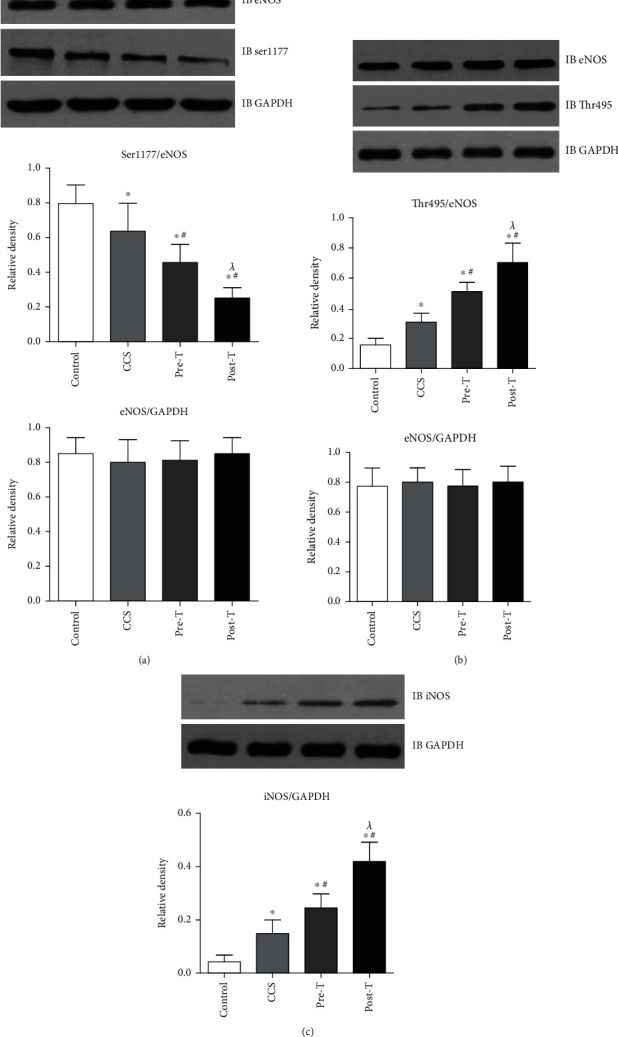
Effects of MPs from STEMI patients with or without thrombolysis on eNOS and iNOS expressions. (a) Compared with those in the controls, MPs from CCS patients and STEMI patients before thrombolysis (pre-T) decreased eNOS phosphorylation at Ser1177. MPs from STEMI patients 2 hours after thrombolysis (post-T) further decreased eNOS phosphorylation at Ser1177. (b) Compared with those in the controls, MPs from CCS patients and STEMI patients before thrombolysis (pre-T) increased eNOS phosphorylation at Thr495. MPs from STEMI patients 2 hours after thrombolysis (post-T) further increased eNOS phosphorylation at Thr495. (c) Compared with those in the controls, MPs from CCS patients and STEMI patients before thrombolysis (pre-T) increased the expression of iNOS. MPs from STEMI patients 2 hours after thrombolysis (post-T) further increased the expression of iNOS. The data are means ± SDs; ^∗^ vs. control; ^#^ vs. CCS; ^*λ*^ vs. pre-T, *P* < 0.05.

The expression of eNOS and iNOS and their phosphorylation were detected by western blotting to investigate the mechanism by which MPs affect vascular function. Compared with MPs from the control group, MPs from CCS and STEMI patients before thrombolysis slightly decreased the level of eNOS phosphorylation at Ser1177 (Figure 4(a)) but increased eNOS phosphorylation at Thr495 (Figure 4(b)) and iNOS expression (Figure 4(c)). Furthermore, the effects on iNOS, eNOS, and its phosphorylation were enhanced by MPs from STEMI patients 2 hours after thrombolysis (Figure 4).

### 3.6. Effects of MPs on Endothelium-Dependent Vasodilatation

**Figure 5:**
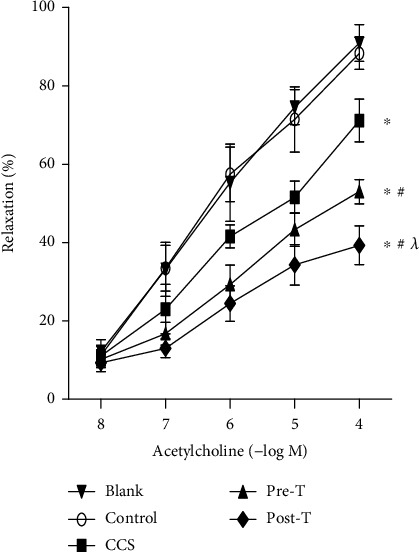
Effects of MPs from STEMI patients with or without thrombolysis on endothelium-dependent vasodilatation. Compared with those in the controls, MPs from CCS patients and STEMI patients before thrombolysis (pre-T) impaired endothelium-dependent vasodilatation. MPs from STEMI patients 2 hours after thrombolysis (post-T) further impaired endothelium-dependent vasodilatation. The data are means ± SDs; ^∗^ vs. control; ^#^ vs. CCS; ^*λ*^ vs. pre-T, *P* < 0.05.

Compared with those in the control group, MPs from CCS and STEMI patients before thrombolysis slightly inhibited endothelium-dependent vasodilatation in isolated aortae (Figure 5). Moreover, the inhibition of endothelium-dependent vasodilatation was enhanced by MPs from STEMI patients 2 hours after thrombolysis (Figure 5). Ach-induced vasodilatation induced by MPs was completely blocked by NG-nitro-L-arginine methyl ester, hydrochloride (L-NAME, a specific inhibitor of eNOS, Sigma-Aldrich) in all groups (data not shown).

## 4. Discussion

This study demonstrated that compared with that in the control group, the concentration of MPs was increased in patients with CCS and STEMI patients with or without thrombolysis (thrombolysis did not further increase MP levels compared with those in STEMI patients before thrombolysis). MPs from patients with CCS and STEMI patients before thrombolysis, especially those from STEMI patients 2 hours after thrombolysis, inhibited the expression of eNOS (immunohistochemistry and western blot analysis of phosphorylation at Ser1177) and the production of NO in the isolated myocardium and vasodilation in vitro and stimulated the expression of iNOS (immunohistochemistry and western blot analysis of phosphorylation at Thr495) and the generation of O_2_^•−^ in the isolated myocardium.

STEMI is feared and valued by medical staff and the general public due to its high mortality rate. Currently, the most effective revascularization method for STEMI patients is PCI. Thrombolysis remains an indispensable measure for effectively improving prognosis and reducing mortality for in STEMI patients who cannot undergo or reject PCI [1–3]. However, ischemia–reperfusion injury after thrombolysis remains a major problem that troubles medical workers [11–13]. The number and function of MPs may be affected by various free radicals (such as alkyl radicals and alkoxy radicals), vasoactive substances (such as leukotrienes and platelet-activating factors), endothelin, and angiotensin produced in response to ischemia–reperfusion [14–16]. MPs can lead to endothelial dysfunction and free radical imbalance, which in turn can stimulate the production of MPs [6–8, 17, 18]. This study showed that compared with those in healthy volunteers (control), the levels of MPs in CCS and STEMI were significantly increased. The increase in MPs may be involved in the occurrence and development of ischemia–reperfusion injury after thrombolysis.

iNOS, which is not expressed in normal tissues, can be stimulated in various pathological states. Overexpression of iNOS results in the production of a large amount of nitric oxide (NO), and excessive NO can combine with free radicals to produce hydroxyl ions and nitrite ions, which can damage endothelial cells and endothelial function [19–21]. Phosphorylation of eNOS at Ser1177, not Thr495, indicates that eNOS is more active, and the activation of eNOS promoted NO generation [22]. Our previous study [23] showed that endothelium-derived microparticles (EMPs) could impair endothelial-dependent relaxation by inhibiting the expression of eNOS and the production of NO. Endothelial cell damage and dysfunction are related to a decrease in NO levels or inactivity, as well as an increase in O_2_^•−^ [24–26], which is also one of the main mechanisms of ischemia–reperfusion injury [14–16]. Our present study showed that MPs from both CCS and STEMI patients decreased NO production and inhibited eNOS phosphorylation at Ser1177 and endothelial-dependent vasodilation. However, the increase in eNOS phosphorylation at Thr495 and O_2_^•−^ generation stimulated iNOS expression in rat myocardial tissue. Moreover, MPs from STEMI patients 2 hours after thrombolysis further enhance these effects compared to those before thrombolysis. The effects of MPs after thrombolysis may lead to the generation of a large number of hydroxyl ions and nitrite ion ions and further damage endothelial function, which may be one of the mechanisms of ischemia–reperfusion injury after thrombolysis.

### 4.1. Limitations of Study

It will be better to collect samples from the distal coronary bed, but the STEMI patients we recruited refused PCI (themselves or by their families); this is one of our limitations. Whether these MPs affect no reflow or increase major adverse cardiovascular events (MACE) was not investigated, and we will continue this research to investigate these indicators.

In summary, changes in the function but not the levels of MPs after thrombolysis may be one of the mechanisms of ischemia–reperfusion injury after thrombolysis. However, since we injected human MPs into rats, there are still immune factors caused by species differences that may affect the reliability of our research results. Therefore, more basic and clinical studies are needed to further validate our research results.

## Figures and Tables

**Table 1:** Clinical characteristics of control (*n* = 20), CCS (*n* = 20), and AMI (*n* = 18).

	Control (*n* = 20)	CCS (*n* = 20)	AMI (*n* = 18)
Age (yr)	48.66 ± 7.41	46.58 ± 9.39	46.58 ± 9.39
Sex (male/female)	10/10	11/9	10/8
TC (mmol/l)	3.32 ± 0.34	3.52 ± 0.63	3.86 ± 0.81^∗^
TG (mmol/l)	1.13 ± 0.37	1.24 ± 0.55	1.77 ± 0.83
HDL (mmol/l)	1.03 ± 0.26	1.11 ± 0.57	1.01 ± 0.66
LDL (mmol/l)	2.38 ± 0.42	2.24 ± 0.96	3.31 ± 0.97^∗^
BMI (kg/m^2^)	20.63 ± 1.25	21.31 ± 1.63	21.19 ± 1.34
Medication antiplatelet	5	20	14^∗^
Statin	8	18	10^∗^

Values are listed as the means ± SDs. TC: total cholesterol; TG: triglyceride; HDL: high-density lipoprotein; LDL: low-density lipoprotein; BMI: body mass index. ^∗^ vs. control and CCS, *P* < 0.05.

## Data Availability

All data were listed in the manuscript.

## References

[1] Mitsis A., Gragnano F. (2021). Myocardial infarction with and without ST-segment elevation: a contemporary reappraisal of similarities and differences. *Current cardiology reviews*.

[2] Partow-Navid R., Prasitlumkum N., Mukherjee A., Varadarajan P., Pai R. G. (2021). Management of ST elevation myocardial infarction (STEMI) in different settings. *International Journal of Angiology*.

[3] Ibanez B., James S., Agewall S. (2018). 2017 ESC guidelines for the management of acute myocardial infarction in patients presenting with ST-segment elevation: the task force for the management of acute myocardial infarction in patients presenting with ST-segment elevation of the European Society of Cardiology (ESC). *European Heart Journal*.

[4] Klinkner D. B., Densmore J. C., Kaul S. (2006). Endothelium-derived microparticles inhibit human cardiac valve endothelial cell function. *Shock*.

[5] Marei I., Chidiac O., Thomas B. (2022). Angiogenic content of microparticles in patients with diabetes and coronary artery disease predicts networks of endothelial dysfunction. *Cardiovascular Diabetology*.

[6] Wang X. L., Zhang W., Li Z. (2021). Vascular damage effect of circulating microparticles in patients with ACS is aggravated by type 2 diabetes. *Molecular Medicine Reports*.

[7] Han W. Q., Chang F. J., Wang Q. R., Pan J. Q. (2015). Microparticles from patients with the acute coronary syndrome impair vasodilatation by inhibiting the Akt/eNOS-Hsp90 signaling pathway. *Cardiology*.

[8] Cheng G., Shan X. F., Wang X. L. (2017). Endothelial damage effects of circulating microparticles from patients with stable angina are reduced by aspirin through ERK/p38 MAPKs pathways. *Cardiovascular therapeutics*.

[9] Hu B., Tian T., Li X. T. (2023). Dexmedetomidine postconditioning attenuates myocardial ischemia/reperfusion injury by activating the Nrf2/Sirt3/SOD2 signaling pathway in the rats. *Redox Report*.

[10] Marsh A., Schiffelers R., Kuypers F. (2015). Microparticles as biomarkers of osteonecrosis of the hip in sickle cell disease. *British Journal of Haematology*.

[11] Schäfer A., König T., Bauersachs J., Akin M. (2022). Novel therapeutic strategies to reduce reperfusion injury after acute myocardial infarction. *Current Problems in Cardiology*.

[12] Ouyang C., Huang L., Ye X., Ren M., Han Z. (2022). Overexpression of miR-1298 attenuates myocardial ischemia-reperfusion injury by targeting PP2A. *Journal of Thrombosis and Thrombolysis*.

[13] Liang H., Li F., Li H., Wang R., Du M. (2021). Overexpression of lncRNA HULC attenuates myocardial ischemia/reperfusion injury in rat models and apoptosis of hypoxia/reoxygenation cardiomyocytes via targeting miR-377-5p through NLRP3/caspase-1/IL-1*β* signaling pathway inhibition. *Immunological Investigations*.

[14] Sun M. S., Jin H., Sun X. (2018). Free radical damage in ischemia-reperfusion injury: an obstacle in acute ischemic stroke after revascularization therapy. *Oxidative Medicine and Cellular Longevity*.

[15] Patzwaldt K., Berezhnoy G., Ionescu T. (2023). Repurposing the mucolytic agent ambroxol for treatment of sub-acute and chronic ischaemic stroke. *Brain Communications*.

[16] Gauberti M., Lapergue B., Martinez de Lizarrondo S. (2018). Ischemia-reperfusion injury after endovascular thrombectomy for ischemic stroke. *Stroke*.

[17] Ci H. B., Ou Z. J., Chang F. J. (2013). Endothelial microparticles increase in mitral valve disease and impair mitral valve endothelial function. *American Journal of Physiology. Endocrinology and Metabolism*.

[18] Lin Z. B., Ci H. B., Li Y. (2017). Endothelial microparticles are increased in congenital heart diseases and contribute to endothelial dysfunction. *Journal of Translational Medicine*.

[19] Cinelli M. A., Do H. T., Miley G. P., Silverman R. B. (2020). Inducible nitric oxide synthase: regulation, structure, and inhibition. *Medicinal Research Reviews*.

[20] Anavi S., Tirosh O. (2020). iNOS as a metabolic enzyme under stress conditions. *Free Radical Biology & Medicine*.

[21] Minhas R., Bansal Y., Bansal G. (2020). Inducible nitric oxide synthase inhibitors: a comprehensive update. *Medicinal Research Reviews*.

[22] Ceylan-Isik A. F., Guo K. K., Carlson E. C. (2009). Metallothionein abrogates GTP cyclohydrolase I inhibition-induced cardiac contractile and morphological defects: role of mitochondrial biogenesis. *Hypertension*.

[23] Ou Z. J., Chang F. J., Luo D. (2011). Endothelium-derived microparticles inhibit angiogenesis in the heart and enhance the inhibitory effects of hypercholesterolemia on angiogenesis. *American Journal of Physiology. Endocrinology and Metabolism*.

[24] Sviridov D., Mukhamedova N., Remaley A. T., Chin-Dusting J., Nestel P. (2008). Antiatherogenic functionality of high density lipoprotein: how much versus how good. *Journal of Atherosclerosis and Thrombosis*.

[25] Zhang Q., Yin H., Liu P., Zhang H., She M. (2010). Essential role of HDL on endothelial progenitor cell proliferation with PI3K/Akt/cyclin D1 as the signal pathway. *Experimental Biology and Medicine (Maywood, N.J.)*.

[26] Sumi M., Sata M., Miura S. (2007). Reconstituted high-density lipoprotein stimulates differentiation of endothelial progenitor cells and enhances ischemia-induced angiogenesis. *Arteriosclerosis, Thrombosis, and Vascular Biology*.

